# Single Sequence Magnetic Resonance Imaging in Serous Otitis Media

**DOI:** 10.7759/cureus.38261

**Published:** 2023-04-28

**Authors:** Kadir Şinasi Bulut, Ahmet Kutluhan, Hüseyin Çetin, Fatih Gul, Mehmet Ali Babademez, Hayati Kale, Mecit Sancak

**Affiliations:** 1 Department of Otolaryngology, Head and Neck Surgery, Haymana State Hospital, Ankara, TUR; 2 Department of Otolaryngology, Head and Neck Surgery, Pamukkale University School of Medicine, Denizli, TUR; 3 Department of Radiology, Yildirim Beyazit University Faculty of Medicine, Ankara, TUR; 4 Department of Otolaryngology, Head and Neck Surgery, Yildirim Beyazit University Faculty of Medicine, Ankara, TUR; 5 Department of Otorhinolaryngology, Private Otorhinolaryngology Clinic, Istanbul, TUR; 6 Department of Otolaryngology, Head and Neck Surgery, Lokman Hekim Hospital, Ankara, TUR

**Keywords:** paracentesis, mastoid cells, tympanometry, magnetic resonance imaging, secretory otitis media

## Abstract

Introduction: We evaluated the usability of short-term (approximately 3 minutes) T2 sequence temporal bone magnetic resonance imaging (MRI) in the diagnosis of serous otitis media (SOM) in our study.

Methods: A prospective study. Otoscopic examination and audiometry-tympanometry were performed on all 73 patients included in the study. All patients underwent short-term T2 sequence temporal bone MRI before the paracentesis procedure.

Results: The mean age of 73 patients (30 female and 43 male) was 7.78 ± 3.01 (3 to 17 years). A total of 134 ear paracentesis operations were performed. As a result of the intraoperative paracentesis procedure, 107 Type B tympanogram and 13 Type C tympanogram were found out of 120 ears that had fluid in the middle ear. Out of 14 ears without fluid flow in the middle ear, five were found to be Type B tympanogram and nine to be Type C tympanogram. The sensitivity of the type B tympanogram in the diagnosis of SOM was 89.1%, the specificity was 64.2%, the positive predictive value was 95.5%, and the negative predictive value was 40.9%. The sensitivity and specificity of short-term T2 sequence MRI in diagnosing SOM were found to be 100% and 100%.

Conclusion:* *Among the available methods, the short-term T2 sequence temporal MRI is the most effective method for evaluating fluid in mastoid cells.

## Introduction

Secretory otitis media (SOM) is one of the most common ear diseases in childhood. Its diagnosis is challenging in children due to the absence of obvious clinical symptoms.

The diagnosis is based on the appearance of vascularized and slightly retracted tympanic membrane on physical examination, conductive hearing loss on audiometry, and type B or type C pressure curves on tympanometry. However, reasons such as sequelae in the eardrum caused by previous middle ear infections and ventilation tube (VT) insertion, abnormal membrane structure after cartilage tympanoplasty, external auditory canal narrowness, and inapplicability of examination and tests in syndromic children bring difficulties to the diagnosis and sometimes even cause misdiagnosis. Due to tympanometry’s low negative predictive value, it can be misleading in some cases [1,2]. In addition, it was observed that in many patients who underwent paracentesis with the diagnosis of SOM did not have fluid in the middle ear [1]. If SOM is not treated, the effusion intensifies over time and many complications such as attic retraction, cholesteatoma, tympanic membrane atrophy, and tympanosclerosis may occur [3]. Therefore, new approaches are needed to overcome the difficulties in the diagnosis of SOM.

For this purpose, we evaluated the usability of short-term (approximately 3 minutes) T2 sequence temporal bone magnetic resonance imaging (MRI) in the diagnosis of SOM in our study.

## Materials and methods

This prospectively designed study was initiated with 78 patients aged between 3 and 18 years, diagnosed with SOM, who applied to the Otorhinolaryngology and Head and Neck Surgery Clinic between January 2018 and April 2019. The study was carried out with 73 patients as five patients discontinued. This study was approved by Yildirim Beyazit University Clinical Research Ethics Committee (approval number: 26379996/04) and conducted in compliance with the Helsinki Declaration, and an informed consent form was signed by the guardians of the patients.

Demographic data of all children were recorded, and otoscopic examination, tympanometry tests and short-term T2 sequence MRI were performed. Patients with normal bone conduction in the audiometry, air-bone path loss, and type B or type C curves in the tympanometry were included in the study. Twelve of 73 patients had unilateral SOM, and a total of 134 ears were evaluated. A temporal MRI was performed two days before tube insertion. In addition, it was noted whether the fluid was obtained as a result of the paracentesis procedure applied to the patients.

Short-term T2 sequence temporal MRI

All MRI examinations were performed by a 3 Tesla MRI device (Magnetom Skyra; Siemens, Germany) using a head coil. T2-weighted axial plane (TR/TE; 1000/136 ms) 3D-SPACE images were used. The field of view was 20-20 cm, the matrix was 380 x 384 pixels, and the slice thickness was 0.5 mm. MRI examinations were re-evaluated using the image archiving and communication system (Extreme PACS, Ankara, Turkey). MRI images were evaluated for the presence of fluid in the middle ear and mastoid cells by an experienced radiologist. No contrast agent, sedation, or general anaesthesia was administered to any patient.

Audiometry-tympanometry

All audiometry tests were performed with the Orbiter 922 version 2 clinical audiometer and tympanometry tests were performed with the 226 Hz OTOflex 100; Otometrics. Conditioned game audiometry was applied especially to patients aged between three and seven years who had adjustment problems. In the audiometric evaluation, airway and bone conduction values were calculated by taking the average of the frequencies of 500 Hz, 1000 Hz, 2000 Hz, and 4000 Hz. The air-bone conduction interval was calculated as the difference between the airway mean and the bone conduction mean. Tympanogram types, pressure, and compliance values were recorded for each patient in tympanometry. Tympanogram values and types were evaluated according to the Jerger classification [4]. Type C1 and Type C2 are designated as Type C.

Statistical analysis

The Statistical Product and Service Solutions (SPSS) (IBM, version 26.0, Armonk, NY) program was used for statistical analysis. Categorical variables were expressed as frequency and percentage (%), and discrete numerical variables were expressed as mean ± standard deviation or median (min. to max.). Shapiro-Wilk analysis of normality was used to evaluate the distribution of the data. In group comparisons, Chi-Square Test and Student's T-Test or Mann-Whitney U test were used to compare categorical and continuous numerical variables, respectively.

Receiver operating characteristic (ROC) analysis was performed to determine the tympanometry pressure value, compliance, and air-bone gap cut-off values, as well as their sensitivity and specificity.

Binary logistic regression analysis was used to determine the effectiveness of tympanometric variables in predicting the presence of fluid in the middle ear. The absence of middle ear fluid was taken as the reference category. The suitability of the modelling was evaluated with the Hosmer and Lemeshow test, Cox & Snell R Square, and Nagelkerke R2 values. A p<0.05 value was accepted as the statistical significance limit.

## Results

The mean age of 73 patients (30 female and 43 male) was 7.78 ± 3.01 (3 to 17 years). SOM was bilateral in 61 patients and unilateral in 12 patients. In the otoscopic examination of all 134 ears, the tympanic membrane Politzer triangle was absent, the membrane was hypervascularized or opaque in some.

The mean bone conduction was 4.74 ± 4.37 (0 to 15) dB; the mean airway was 32.13 ± 8.29 (13 to 53) dB, and the air-bone gap was 27.38 ± 6.32 (13 to 40) dB in the audiometry. The mean pressure value was -267.01 ± -88.80 (-474 to -102) daPa, and the mean compliance value was 0.16 ± 0.12 (0.01 to 0.48) mL in the tympanometry. Tympanometry pressure values were calculated as -292.01 ± -73.89 daPa for Type B and -139.73 ± -27.92 daPa for Type C according to tympanogram types. Demographic and clinical data of the study groups are shown in Table 1.

**Table 1:** Demographic and clinical characteristics of the patient group SOM: serous otitis media; daPa: decapascals

SOM group (n=134)			
		n	Mean ± SD
Age, y		-	7.78 ± 3.01
Sex, n			
	Female	30	-
	Male	43	-
Pure tone audiometry			
	Airway	-	32.13 ± 8.29
	Bone canal	-	4.74 ± 4.37
	Air-bone gap	-	27.38 ± 6.32
Tympanometry pressure value, daPa		-	-267.01 ± -88.80
	Type A	-	
	Type B	112 (83.6%)	-292.01 ± -73.89
	Type C	22 (16.4%)	-139.73 ± -27.92
Tympanometry compliance value, mL		-	0.16 ± 0.12

The mean tympanometry pressure values ​​of 120 ears with fluid obtained in the middle ear and mastoid cells in T2 sequence MRI were -275,43 ± -86.49 daPa (-474 to -102), while the mean for the group with fluid in only mastoid cells was -194.79 ± -76.98 daPa (-343 to -109). There was a significant difference between tympanogram types. Type B tympanogram was found in 107 ears, and Type C tympanogram was found in 13 of 120 ears with fluid in the middle ear and mastoid cells, while Type B tympanogram was obtained in five and Type C tympanogram in nine of those with fluid in only mastoid cells.

The mean compliance value was found to be 0.15 ± 0.11 (0.01 to 0.48) mL in patients with fluid in the middle ear and mastoid cells, and 0.28 ± 0.14 (0.02 to 0.48) mL in patients with fluid in only mastoid cells which was significantly different. In all 120 ears with fluid in the middle ear and mastoid cells observed on MRI, fluid flow was observed during intraoperative paracentesis. However, no fluid flow was observed during paracentesis in the 14 ears in which only fluid was detected in the mastoid cells on MRI (Table 2).

**Table 2:** Relationship between fluid presence in MR images and tympanogram and paracentesis status of SOM patients SOM: serous otitis media; daPa: decapascals; MRI: magnetic resonance imaging

		Presence of fluid in T2 sequence MRI		p
		Middle Ear + Mastoid (n=120)	Mastoid (n=14)	
Tympanometry, n				
	Type B	107	5	<0.001
	Type C	13	9	
Tympanometry pressure value, daPa		-275.43 ± -86.49	-194.79 ± -76.98	0.002
Tympanometry pressure value, daPa		0.15 ± 0.11	0.28 ± 0.14	0.006
Presence of fluid in paracentesis				
	Yes	120	0	<0.001
	No	0	14	

While 107 of 120 ears with fluid drainage in paracentesis were Type B and 13 were Type C, five of 14 ears without fluid flow were found to be Type B and nine as Type C tympanogram. Considering the tympanometry pressure values, the mean of those with fluid drainage was -275.43 ± -86.49 daPa (-474 to -102), and -94.79 ± -76.98 daPa (-343 to -109) of those without drainage which was significantly different (Table 3).

**Table 3:** The relationship between tympanogram results and paracentesis status of the SOM group SOM: serous otitis media; daPa: decapascals

		Presence of fluid in paracentesis		p
		Yes	No	
Tympanometry (n)				
	Type B	107	5	<0.001
	Type C	13	9	
Tympanometry pressure value, daPa		-275.43 ± -86.49	-194.79 ± -76.98	0.001
					Tympanometry compliance value, mL		0.15 ± 0.11	0.28 ± 0.14	0.006

**Figure 1:**
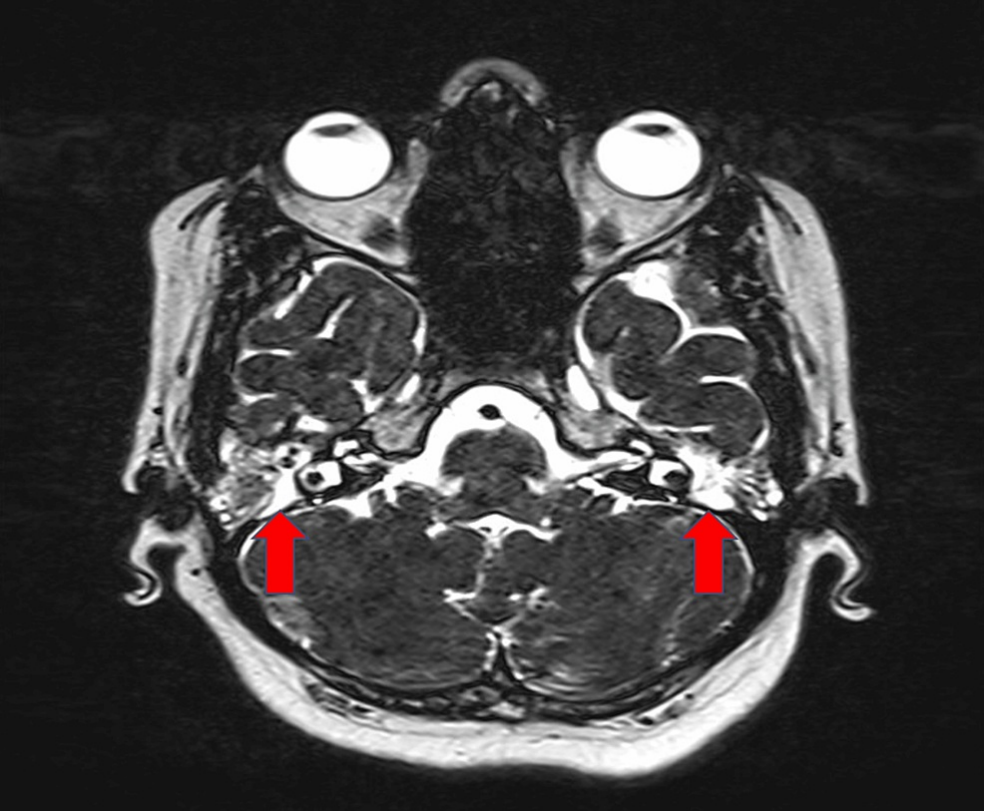
Fluid presence in middle ear on preoperative short-term T2 sequence MRI MRI: magnetic resonance imaging

**Figure 2:**
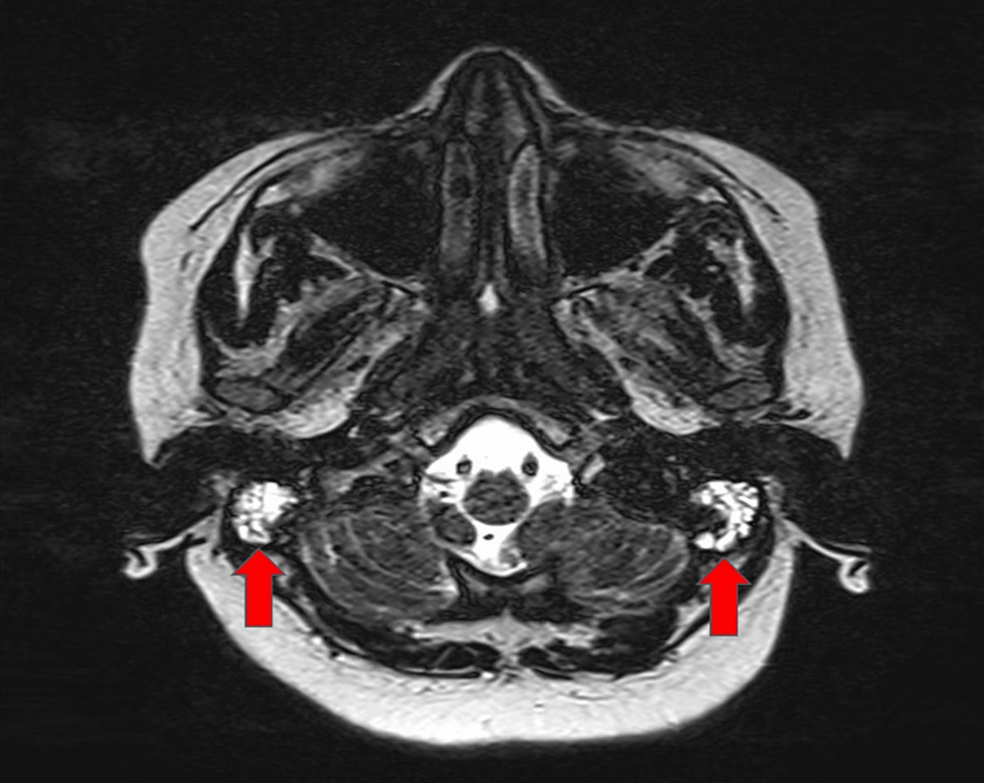
Fluid presence in mastoid cells on preoperative short-term T2 sequence MRI MRI: magnetic resonance imaging

There was increased density in the short-term T2 sequence MRI in mastoid air cells of 134 ears and in the middle ear cavity of 120 ears which were compatible with fluid presence (Figure 1 and Figure 2).

**Figure 3:**
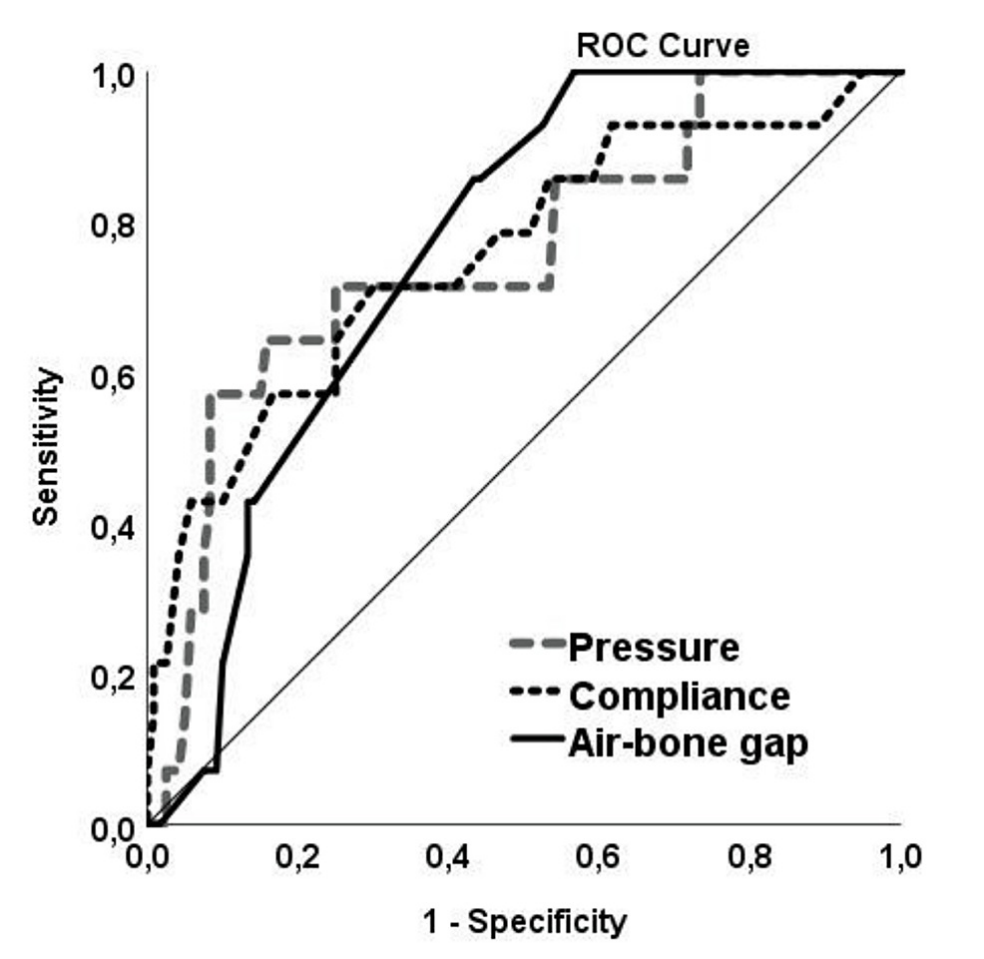
ROC analysis of tympanometry pressure and compliance, and air-bone gap values in the diagnosis of SOM ROC: receiver operating characteristic; SOM: serous otitis media

In ROC analysis, tympanometry pressure was found to have 71.7% sensitivity and 75% specificity at a cut-off value of -218.5 daPa (AUC: 0.755; p=0.002) in SOM evaluation (Figure 3). For tympanometry compliance at 0.185 mL cut-off value, 71.4% sensitivity and 70% specificity (AUC: 0.757; p=0.002) were determined. In the audiogram, 85% sensitivity and 57% specificity were found for the air-bone gap at a cut-off value of 25.50 (AUC: 0.760; p=0.001) (Figure 3).

The sensitivity of the Type B tympanogram in the diagnosis of SOM was 89.1%, the specificity was 64.2%, the positive predictive value was 95.5%, and the negative predictive value was 40.9%. The sensitivity and specificity of short-term T2 sequence MRI in diagnosing SOM were found to be 100% and 100%.

Binary logistic regression analysis was performed to evaluate the effectiveness of tympanometric variables in predicting the presence of fluid in the middle ear. Accordingly, a significant model including tympanometry pressure and the air-bone gap in pure tone audiometry was obtained (p<0.001). The tympanogram pressure in this model had a significant effect (OR: 0.990; 95% CI: 0.982-0.998; p=0.019) while the same significance was not found in the air-bone gap (OR: 1.100; 95% CI: 0.995-1.217; p=0.062). The Cox & Snell R Square and Nagelkerke R2 values for constructed model were 0.134 and 0.275, respectively. In addition, it was determined that the modelling did a good fit according to the Hosmer and Lemeshow test (p=0.678).

## Discussion

Antibiotics, decongestants, steroids, and antihistamines are the first-line non-surgical treatment approaches for SOM, which is a more common disease in childhood [5-8]. Although the use of non-surgical treatments is a controversial issue in the literature, there is no consensus on how and for how long they should be applied. In some studies, it is stated that antihistaminic and decongestant treatments are not effective [6,9]. In addition, there are studies indicating that antibiotherapy is beneficial only in the short term with low effectiveness. Guidelines have been prepared for the SOM treatment protocols [9,10]. The SOM Clinical Practice Guide was published by a committee which consists of the American Academy of Otolaryngology-Head and Neck Surgery, the American Academy of Pediatrics, and the American Academy of Family Physicians in 2004 and was updated in 2016 [9-11]. Accordingly, decongestants and antihistamines have no place in treatment, and steroid therapy and antibiotherapy should not be used in routine practice due to their lack of long-term effectiveness. The committee does not suggest a path to follow for non-surgical treatments, since there is no non-surgical treatment with proven efficacy [10]. Surgical treatment is recommended in children with erosive middle ear anatomical structures or tympanic membrane, hearing loss for four months or more, and developmental delay independent of hearing loss and duration.

The primary option in surgical treatment is the VT application [10]. Although VT as a surgical method is not a strenuous procedure, there are some reluctances regarding this application, such as the spontaneous resolution of SOM, premature loss of the VT, need for repositioning, and infiltration of microorganisms or allergens [12]. In addition, complications such as foreign body reaction due to VT application, VT falling into the middle ear, tympanic membrane retraction or perforation, cholesteatoma formation, permanent or temporary hearing loss, granulation tissue formation, VT occlusion, and tympanosclerosis have been shown in the literature [13-17]. Moreover, it is known that in some cases paracentesis yields no fluid efflux [1,2,18,19]. For these reasons, it is thought that a rational approach would be to correctly apply non-surgical treatment options for sufficient time before deciding on surgical treatment in SOM patients [10]. To this end, we evaluated T2 short sequence MRI in the diagnosis and follow-up of SOM to prevent unnecessary surgical interventions.

Although there are different approaches in terms of treatment protocols, SOM is a disease that needs attention due to its complications and effects on quality of life. Intermittent recurrences of SOM decrease the quality of life and increase the risk of complications. SOM can be diagnosed with otoscopic examination and audiological tests but it is challenging to diagnose SOM in some cases such as patients with narrow external auditory canal, effusion due to cartilage tympanoplasty, and otitis sequelae such as calcareous plaque in the eardrum, as well as in syndromic children. MRI can be advantageous and practical in evaluating the fluid in the middle ear cavity, especially in these patients.

In our study, we evaluated the performances of tympanometry pressure value, compliance value, and the air-bone gap in the diagnosis of SOM. Accordingly, pressure and compliance values had similar performance. The sensitivity of the air-bone path gap was higher, and the specificity was lower than that of the tympanogram.

Since audiometric evaluation in children cannot always be performed under optimum conditions due to cooperation problems, the Auditory Brainstem Response (ABR) test is preferred instead of audiometry. In our study, we found a higher rate of positive predictive value of Type B curves in tympanometry. The diagnostic performance values we obtained were compatible with the literature [1,2].

In clinical practice, paracentesis and aspiration procedures are accepted as the gold standard methods for the detection of middle ear fluids. We found that tympanogram pressure is an independent factor in estimating the presence of fluid in the middle ear. However, our study shows that short-term T2 sequence MRI, which is fluid-indexed, has 100% sensitivity in detecting fluid in the middle ear or mastoid cells, and can be a valuable non-surgical method for use in the diagnosis of SOM.

Since the most common indication for VT application is considered to be permanent and unresponsive to antibiotherapy serous or secretory otitis media, otoscopic examination appears to be the most valuable diagnostic tool we have. However, there is no clear criterion for determining the appropriate indication for suspected cases. Our study suggests that tympanometry pressure monitoring can also be used as an adjunct criterion in the otoscopic examination for tube insertion. Observing a standard course in the serial tympanogram pressure data in the follow-up may bring a perspective towards avoiding surgery, despite the presence of serous fluid in the otoscopic examination.

In some studies, it has been shown that there is less recurrence in patients with good mastoid aeration [20,21]. Since the tympanic membrane has a flexible structure that allows the changes between the middle ear and the ambient pressure, tympanic membrane movement causes volume changes in the middle ear and mastoid cells. The tympanic membrane is more affected by pressure changes when the mastoid cell volume is small [22]. Fluid formation in mastoid cells may reduce mastoid cell volume, exposing the tympanic membrane to enormous pressure changes and causing retraction.

The distance at which gas exchange takes place between the blood vessels and the basement membrane of the mastoid cell mucosa is an average of 40 μm [22]. Inflammation in the mastoid cell mucosa increases the number and diameter of blood vessels [23]. This causes an increase in gas exchange and an increase in the physiological negative pressure extending to the middle ear, thereby disrupting the balance. The higher the degree of disruption of transmucosal gas exchange, the greater the reduction in middle ear total pressure [24]. Fluid accumulation in mastoid cells also increases inflammation and changes the middle ear pressure by causing negative pressure. Cinamon et al. emphasized that the eustachian tube works together with the mastoid cells and provides aeration of the middle ear [25]. Inflammation in mastoid cells can be detected as thickening of the mastoid cell mucosa and fluid accumulation on MRI.

The acquisition of MRI scans requires patients to remain still throughout the imaging session. This requirement may pose a challenge for pediatric patients, particularly younger children, who may have difficulty complying with the protocol. Therefore, the present study is limited by the potential difficulties associated with conducting MRI scans in pediatric populations. Furthermore, although the imaging process is typically brief, children may also struggle to adjust to the restricted space within the MRI scanner.

MRI is known to be an expensive method. In our study, the cost of T2 sequence temporal MRI, which takes approximately one-fourth of the time required by a standard temporal MRI procedure, is remarkably reduced. A standard temporal MRI procedure costs $33.75, while a T2 sequence MRI costs $19.68. Audiometry and tympanometry cost $16.50 per patient and have a similar cost to T2 sequence MRI. However, when the diagnosis of SOM in the 73 patients included in the study was evaluated with T2 sequence temporal MRI, surgery would not be required in nine patients. Considering that the cost of the surgical procedure is $89.10 for each patient, a T2 sequence MRI is considerably less expensive. In addition, the costs that may arise due to the intervention to complications related to the surgical procedures can be avoided.

## Conclusions

The function of mastoid cells together with the eustachian tube is crucial in middle ear aeration. Among the available methods, the short-term T2 sequence temporal MRI is currently the most effective method for evaluating fluid in the mastoid cells, and it can be used as an adjunctive examination in the management of SOM.

In patients with tympanogram Type B or Type C where the decision of VT insertion cannot be taken due to the inability to detect the fluid present in the otoscopic examination in serial follow-up, short-term T2 sequence temporal MRI can be an effective method in deciding for surgical indication.
